# Teacher’s perspective regarding Co-teaching Classes: A qualitative study

**DOI:** 10.12669/pjms.40.11.9258

**Published:** 2024-12

**Authors:** Afreenish Malik, Brekhna Jamil, Maidha Jadoon, Nowshad Asim

**Affiliations:** 1 Afreenish Malik, BDS, MPHE Lecturer, Institute of Health Professions Education & Research (IHPER), Khyber Medical University, Peshawar, Pakistan; 2 Brekhna Jamil, BDS, MPH, MHPE, PhD Director, Institute of Health Professions Education & Research (IHPER), Khyber Medical University, Peshawar, Pakistan; 3 Maidha Jadoon, BDS, MPHE Lecturer, Department of Medical Education and Research (DMER), Women Medical College, Abbottabad, Pakistan; 4 Nowshad Asim, MBBS, DFM, MHPE Lecturer, Institute of Health Professions Education & Research (IHPER), Khyber Medical University, Peshawar, Pakistan

**Keywords:** Co-teaching, Teacher’s perspectives, Healthcare education

## Abstract

**Objective:**

To explore teacher’s perspectives regarding co-teaching classes.

**Methods:**

Online interviews of participants were conducted through Zoom meetings at a suitable time the researcher and the participant decided beforehand. Teachers’ perspective regarding co-teaching classes were studied. The study design was qualitative, and phenomenological. A purposive sampling technique was opted in which participants involved in co-teaching in the field of medicine for not less than one year were chosen. Data was collected through a semi structured interview, with nine open ended question, after validation from experts.

**Results:**

Thematic analysis of the data was done in three phases. Fifty-three codes were identified from the transcriptions of the interviews in the initial coding. Twelve codes were derived in axial coding and finally, five themes were formed. Overall teachers showed a very positive response towards co-teaching. They discussed a lot of benefits of co-teaching. The opportunities and challenges in the co-teaching classes were also discussed. and most importantly the interaction of the co-teachers in the planning phase of the session, during the class and in the assessment were explored.

**Conclusion:**

Teachers described their experience of co-teaching as productive, pleasant, and enriching. The professional growth of teachers is fostered through co-teaching. Collaborative harmony develops among co-teachers. Teacher’s satisfaction also increases by comprehensive knowledge transfer. Although some challenges were faced by teachers in co-teaching classes. The most common challenge of co-teaching was time management. Dominating nature or irresponsible teachers was also a challenge to deal with.

## INTRODUCTION

Co-teaching can be defined as two professionals teaching the same course to a group of pupils in a joint space.^1^ Co-teaching is known by different terms such as team teaching, shared teaching, and collaborative teaching and can be modified in different contexts. In literature, Cook and Friend discuss five co-teaching models: One teaching, one assisting, station teaching, parallel station, alternative teaching, and team teaching. These models are differentiated based on the roles that each teacher is given throughout a lesson. The description of these models is mentioned in Table-I. The one-teaching, one-assisting model is the most adopted because of its ease of implementation.^2^ In this study (according to Table-I) team teaching model was the focus but is generally referred to as co-teaching.^3^ Co-teaching is said to be quite time-consuming and demanding. Teachers must plan, prepare, and deliver the content together and assess it.^4^

**Table-I:** Search Strategies.

Sr. No.	Term	Google Scholar	PubMed	BMC
1	Co-teaching	58,000	62	50
2	Teachers Perspectives	5200000	7213	7387
3	Healthcare Education	6200000	455766	58529
4	Co-teaching and medical education	18800	28	33
5	1 & 2	29300	5	19
6	1 & 3	8450	9	28
7	((co-teaching) AND (teachers view)) AND (medical education)	2,880,000	15	2103
8	Teachers’ perspective on co-teaching	43,300	142	9
9	((co-teaching) AND (teachers perspectives)) AND (healthcare education)	18500	29	789

However, Weiss’s study concluded that the teacher’s teaching styles were essential in co-teaching. In a study, crow and Smith first assumed that co-teaching would diminish the autonomy of the teacher, but they concluded that collaboration had a positive effect on students and teachers both. Today, it is believed that learning is more of a social than an individual process; students gain more knowledge from conversations and group problem-solving than from responding to teacher-produced information.^5^ The Flexner’s approach was followed for years in health education, but due to its limitations, an integrated curriculum was introduced to overcome the limitations.^6^

Many medical schools opt for the integrated curriculum very rapidly due to its compelling nature, but it is pretty resourceful, and many couldn’t adopt it. Co-teaching as an alternative to an integrated curriculum has been studied. Two instructors with fundamental and clinical education expertise jointly taught the applied and basic principles. As a result, the pupils’ comprehension and retention of the material improved.^7^ Co-teaching can be used as a tool for the smooth delivery of an integrated curriculum. 8

**Fig.1:**
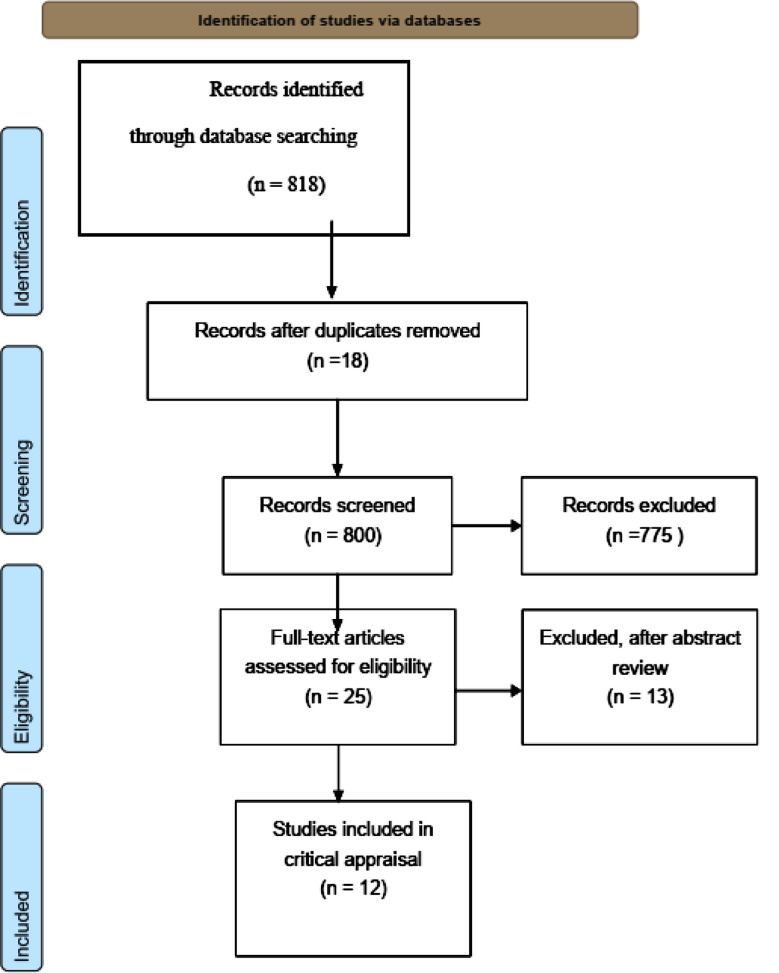
Prisma flow diagram.

### PRISMA Flow Diagram:

“PRISMA” stands for Preferred Reporting Items for Systematic Reviews and Meta-Analyses. The flowchart is shown below in compliance with PRISMA guidelines. When selecting the articles, the inclusion and exclusion criteria were considered (Table-II Critical Appraisal).

**Table-II:** Critical Appraisal.

S. No	Author/Year	Title	Research type	Key findings
1.	Afsaneh Dehnad, Maryam Jalali, Saeed Shahabi, Parviz Mojgani and Shoaleh Bigdeli 2021	Students’ view on supportive co-teaching in medical sciences: a systematic review (Dehnad et al., 2021)	Systematic review	• Co-teaching is an innovative teaching method developing in a collaborative culture. The reviews of all the research showed that the students had favorable opinions of SCT • Students were more involved in learning and better understood the connection between basic and clinical sciences. • The simultaneous presence of the two teachers also gave students an excellent example of inter-professional interaction and the communication skills they would need in everyday life. • Some research raised issues and worries about SCT, such as personality conflicts and a lack of coordination among the instructors, making the class distracting, perplexing, or even upsetting.
2.	Sadhana Sharma, Ravikirti, Asgar Ali, Roshan Takhelmayum, Mala Mahto, Rathish Nair 2017	Co-teaching: Exploring an Alternative for Integrated Curriculum (Sharma et al., 2017)		• This study was an attempt to investigate whether integrated co-teaching could be used to fill the gap left by the absence of an integrated curriculum. • Co-teaching may be used in place of an integrated curriculum because it promotes comparably higher information retention.
3.	Joanne M Willey, Youn Seon Lim, Thomas Kwiatkowski 2018	Modeling integration: co-teaching basic and clinical sciences medicine in the classroom (Willey et al., 2018)		• Results indicate that students value the integration of basic and clinical sciences through co-teaching. However, deliberate preparation and faculty cooperation are needed for interactive co-teaching.
4.	Jayne Crow & Lesley Smith 2003	Using co-teaching as a means of facilitating interprofessional collaboration in health and social care (Crow & Smith, 2003)		• The statistics shed light on how co-teaching can demonstrate shared learning and teamwork in the classroom and emphasise adequately structuring co-teaching interactions, including humour, tension, varied knowledge bases, and argument techniques. • We established an active learning environment through the purposeful utilisation of the interactions made possible by coteaching.
5.	Kenneth Tobin 2014	Twenty Questions about Co-teaching (Pitts et al., 2014)		• By starting co-teaching, the teaching process was looked at through new theoretical lenses. Teachers observed and gained knowledge from one another’s instruction; in other words, they developed their teaching skills by sitting at one another’s elbows.
6.	Jay D Orlander, Mukund Gupta, B Graeme Fincke, M Elizabeth Manning & Warren Hershman	Co-teaching: a faculty development strategy (Orlander et al., 2000)		• A preliminary orientation is followed by three iterative teaching, evaluation, and planning phases in the coteaching paradigm. The co-teachers analyse the teaching interaction during the debriefing phase. Planning involves practising the insights learned during debriefing to prepare for the upcoming teaching interaction.
7.	Connie R Shi, Jasmine Rana and Susan Burgin	Co-teaching: applications in medical education (Shi et al., 2018)		• The dynamic character of co-teaching makes it a proper teaching method for teachers and students. • Five different co-teaching models have been mentioned and are as follows: Team teaching, parallel teaching, one teach, one support, alternative teaching, station teaching, and its advantages, drawbacks, and use in medical education are covered.
8.	Henri V. Pesonen, Anna Rytivaara, Iines Palmu and Anna Wallin 2019	Teachers’ Stories on Sense of Belonging in Co-Teaching Relationship (Pesonen et al., 2021)		• This article examined what influences teachers’ sense of belonging in their co-teaching partnerships and what doesn’t. • The three elements of teachers’ work practices, interpersonal relationships, and personality traits were used to generate a sense of belonging among co-teachers.
9.	Jennifer Lock, Tracey Clancy, Rita Lisella, Patricia Rosenau, Carla Ferreira, Jacqueline Rainsbury 2016	The Lived Experiences of Instructors Co-teaching in Higher Education (Lock et al., 2017)		• Out of the study, five themes were identified. 1.First, the instructors define the co-teaching process’ essential components. 2.Second, we investigate the influence of prior professional connections on forming the co-teaching relationship. 3.The qualities necessary to foster a collaborative instructional partnership are then examined. 4.Fourth, the lecturers discuss their insights on what they discovered while co-teaching and from one another. 5.Fifth, difficulties with co-teaching are mentioned, and strategies for overcoming them are presented.
10.	Lyailya Shakenova 2017	The Theoretical Framework of Teacher Collaboration (Shakenova, 2017)		• Through exchanging knowledge and ideas, collaboration benefits teacher learning, improving students’ performance. • Teacher collaboration lessens instructors’ workload and gives their coworkers moral support. Collaboration, however, might decrease teachers’ motivation and lessen their sense of autonomy in their teaching. • Barriers to teacher collaboration were identified as time, school culture, and micropolitics. Respect, trust, shared understanding, shared aims with coworkers, and positive attitudes toward teaching were all mentioned as elements fostering teacher collaboration.
11.	Katrina Arndt Jeffrey Liles 2014	Pre-service Teachers’ Perceptions of Co-teaching: A Qualitative Study (Arndt & Liles, 2010)		• Two conclusions were made: oFirst, preservice instructors had reservations about the procedure but were open-minded about cooperative teaching. oSecond, preservice instructors viewed their distinct areas as "separate spheres" in their thinking. • The study’s findings imply that teacher preparation programs should consider how their organisations’ covert functions could undermine their efforts to instil in students the attitudes and behaviours that will make them successful classroom teachers.
12.	Lishan Yang & Preman Rajalingam 2019	Are Two Teachers Better than One? Team Teaching in TBL (Yang & Rajalingam, 2020)		• Team-based learning was incorporated in the first two years of the undergraduate curriculum. For which co-teaching was used, it involved one subject expert and one facilitator to run the TBL session.

### Critical appraisal:

Co-teaching is defined as two educators working together with groups of learners and sharing the planning, organization, delivery, and assessment of instruction, as well as the physical space.^9^ Co-teaching theoretically mirrors social constructivist learning theories, emphasizing participant engagement and social learning. Thus supporting Vygotsky’s sociocultural theory of learning and Bandura’s social learning theory.^10^ Co-teaching is a flexible teaching method that may be adjusted to various contexts and learning demands. Cook and Friend define five co-teaching models: Team teaching, Parallel teaching, One teaching, one support, Alternative teaching, and Station teaching.^11^ Co-teaching has benefits for teachers as well as the students. Enhancing students’ performance and promoting the professional growth of staff . Students get multiple perspectives on a single topic, thus broadening their views. Another impact of bringing multiple perspectives is learning to respect others’ opinions.^12^

Some studies suggest that co-teaching helps reduce the workload, generates a sense of responsibility in teachers, provides various learning opportunities to teachers, and is also helpful for self-reflection.^7^ Some students have found co-teaching distracting and disturbing; as earlier discussed, a practical co-teaching session requires a lot of planning, and if not done, it results in a disorganized session.^13^ Students most reported complaint was about the confusion of which teacher they should go to. Grading of homework by two teachers developed quite a pressure on the students. Teachers found co-teaching a lot time-consuming and challenging.^14^ The idea of an integrated curriculum stepped in which the students learn the basics and incorporate them into the clinical setting, resulting in greater understanding. This also promotes students’ ability to think like doctors from starting medical school.^15^

However, as integration demands resources, every medical school can’t implement it. A study was done to use the co-teaching as an alternative to the integrated curriculum. The basic concepts and the applied component were co-taught by two teachers with basic and clinical backgrounds, respectively.^16^ In literature, teachers have always shown an optimistic viewpoint on co-teaching. The most common comment from teachers regarding their experience with co-teaching was that they learned quite a lot in the process It helped them gain insight into their strengths and weaknesses by reflecting upon their work.^17^ There were merely negative comments from teachers while reflecting on their co-teaching experience. However some of the drawbacks included time-consuming planning sessions not agreeing on one point with the co-teacher understanding and managing the co-teaching process.^18^

Although there has been little research on teachers’ perspectives in medicine, it deviated from the conventional teaching model and is still rather significant. Despite existing research on co-teaching in healthcare education, there is limited understanding of teachers’ perspectives on supportive co-teaching classes. This study aimed to explore these perspectives, addressing the challenges faced by teachers in co-teaching environments. By identifying these challenges, the study will provide valuable insights for future research and help develop strategies to overcome the obstacles in co-teaching, ultimately enhancing the effectiveness of healthcare education.

### Search strategy:

The literature was searched using Google Scholar, PubMed, Medline, The Clinical Teacher, and BMC databases to find articles relevant to the study’s objectives. Boolean operators (AND, OR) were used to refine the search.

### Inclusion criteria:

- Full-text journal articles.
- Articles written in English.
- Original articles, insights, systematic reviews, and review articles.
- Studies conducted worldwide.

### Exclusion criteria:

- Duplicate articles.
- Non-specific articles.
- Letters to the editor.
- Blogs.
- Short communications.
- Case reports.

## METHOD

The study was conducted at Khyber Medical University Institute of Health Professions Education and Research, Peshawar over six months.

### Ethical Approval:

The study was approved by the Ethics Committed of the institution. Ref. 1-11/IHPER/MHPE/KMU/23-42, Dated: 16-06-2023.

The study design was a qualitative phenomenological study. Purposive sampling was adopted, and data was collected through a semi-structured online interview based on open-ended validated questions. 5-8 participants (teachers), depending upon data saturation. The sample size in qualitative study depends upon data saturation. Thus, the sample size in our study was also decided upon data saturation. For that, the interviewed eight participants fulfilled the eligibility criteria of our study. Further, more participants were interviewed till data saturation was obtained. The audios were transcribed, and themes were generated after open and axial data coding. Ethical and quality issues were ensured. The ethical approval number was 23-42.

## RESULTS

### Demographic Details):

The study participants’ professional and demographic details are displayed in the Table-III regarding their experiences of co-teaching. Unique codes (such as F1, Z2) are used to identify the participants, and the information includes their gender, credentials, and years of experience co-teaching. There were four men and four women among the participants in the table, representing a mix of genders. Members of the Royal College of Surgeons (MRCS), FCPS (Fellow of the College of Physicians and Surgeons), MBBS (Bachelor of Medicine, Bachelor of Surgery), MHR (Master of Health Research), MHPE (Master of Health Professions Education), and CHPE (Certificate in Health Professions Education) are just a few of the participants’ varied backgrounds. One of the participants was a Bachelor of Dental Surgery or BDS. The participants’ years of co-teaching experience span from 2 to 15 years, showing a significant diversity in the length of time they have been involved in co-teaching activities. Overall, the table highlights the qualifications and degree of co-teaching experience of the study participants, giving an overview of their diverse backgrounds and experiences.

**Table-III:** Demographic Details.

Participants	Gender	Qualification	Co-teaching experience (years)
Fl	Male	MBBS, FCPS	4
Z2	Female	MBBS, FCPS, MRCS, MHPE	5
T3	Male	MBBS, FCPS	11
A4	Male	MBBS, FCPS, MHR, CHPE	14
PS	Female	MBBS, FCPS	15
M6	Female	MBBS, FCPS	3
A7	Male	MBBS, FCPS, MHR	14
AS	Female	B DS, MHPE	2

First, the interviews were transcribed. The initial coding cycle was done manually; 53 codes were derived from the transcriptions, and the open coding was complete. Then, the already defined codes from the first cycle were analyzed for axial coding, and the linking codes were grouped under a broad code. A total of 12 codes were made in the axial coding, which was the second coding cycle. The codes from the second cycle were again linked with the matched ones and were grouped under a theme (Table -IV).

**Table-IV:** Codes from open & axil coding.

Codes from open coding	Codes from axial coding	Themes
Problem-Solving Skills	Usefulness of CT	Co-Teaching Impact
Professional Development		
Teacher Satisfaction (by comprehensive teaching)		
Passive Learning of Teachers		
Punctuality increases		
Sense of responsibility increases		
Effective Learning Environment		
Better Results		
Help Available for micromanagement		
Unique		
Student		
Engagement Productive		
Time-constraint	Downside of CT
Planning requires time and effort	
Differing teaching styles	
Dominating nature of teacher	
Little experience of new teachers	
Irresponsible teachers	
Predefined Assessment	
Unfamiliar to Co-teaching	
Concept Clarification	Adding value to the program	Enhanced Learning
Effective learning environment		
Productive		
Multiple Perspectives		
Better Results		
Problem-Solving Skills		
Multiple perspectives	Coherent knowledge transfer	
Inquiry-Based Learning		
Better Results		
Effective learning environment		
Concept Clarification		
Collaboration	Collaborative synergy	Collaborative Harmony
Useful planning sessions		
Task Divided by teachers		
supplementing each other		
Modelling Collaboration		
Back-Up support		
Help Available for micromanagement		
Harmony		
Flexibility		
Pleasant	Co-teachers bond	
Good		
Enriching		
Wonderful		
Positive		
Satisfying		
Confidence		
Productive		
Harmony		
Teacher Satisfaction (by comprehensive teaching)	Teachers contentment	
Comprehensive Teaching		
Productive		
Confident		
Interactive lectures	Dynamicity	Dynamic Teaching
Small Group Discussions/demonstrations		
Active Learning Strategies		
Invite co-teacher		
Multiple perspectives		
Student Engagement		
Modelling Collaboration		
Shared Responsibilities		
Collaboration		
Interactive session		
Interactive lectures	Teaching Techniques	
Small Group Discussions/demonstrations		
Active Learning Strategies		
Flipped Classroom		
Inquiry-Based Learning		
Two teachers teach together in the same class		
Differing teaching styles		
Punctuality increases	Sense of responsibility	Collaborative Growth
Invite co-teacher		
Shared Responsibilities		
supplemeting each other		
Task Divided by teachers		
Back-Up support		
Mutual decision for critical students		
Sense of responsibility increases		
Professional Development	Professional growth	
Punctuality increases		
Sense of responsibility increases		
Passive Learning of Teachers(gestures)		
Useful planning sessions		
Two teachers teach together in the same class		
Flexibility		
Assessment is taken together.	Management skills	
Problem-Solving Skills		
Predefined Assessment		
Small Group Discussion		
Collaboration		

Co-teaching, a unique teaching strategy, impacts the students and teachers. When teachers were addressed about the opportunities and challenges that occurred to them in co-teaching sessions, they presented with a wide range of perspectives in which the opportunities were more than the challenges. The opportunities included the professional development of the teachers as throughout the session, passive learning of the teacher is carried out by learning from their co-teacher. Together with the co-teacher, the classroom is managed in all contexts, whether teaching, answering queries, managing group activities, or checking for micromanagement of the class, thus enhancing problem-solving skills and increasing the teachers’ sense of responsibility. Teaching becomes comprehensive, and clear concepts are delivered to students with no ambiguity left behind (Z2).

The teachers’ bond is essential in co-teaching, as two teachers plan, run, and assess the class together. When participants were asked about their interaction with the co-teacher, there were only positive responses. The data analysis was performed by first transcribing the interviews. The initial coding cycle was done manually; 53 codes were derived from the transcriptions, and the open coding was complete. Then, the already defined codes from the first cycle were analyzed for axial coding, and the linking codes were grouped under a broad code. A total of 12 codes were made in the axial coding, which was the second coding cycle. The codes from the second cycle were again linked with the matched ones and were grouped under a theme. At this stage, theme formation was achieved. Five themes were formed.

## DISCUSSION

In the rapidly changing trends in medical education, the integrated curriculum and co-teaching are modern introductions. The following are the four essential elements of co-teaching: delivering relevant education to a varied population of students, having two or more instructors, and using standard settings.^19^ The interconnected themes related to co-teaching, which are Co-Teaching Impact, Enhanced Learning, Collaborative Harmony, Dynamic Teaching, and Collaborative Growth, lead to an effective and productive environment and better outcomes for teachers and students. In discussing the co-teaching impact, the positive and negative impacts were discovered for teachers primarily. It positively influenced teachers by fostering professional development, problem-solving skills, and shared responsibility.

At the same time, the downside of co-teaching concerning teachers included requiring a lot of effort and time in the planning and conducting the session. This challenge of co-teaching was discussed in the literature, too.^20^ Enhanced learning occurs by getting multiple perspectives regarding a topic; the concept is well made and clarified, and teachers gain satisfaction by delivering this comprehensive knowledge. Teaching dynamics are improved by collaborative planning, classroom management, and resource use. It also included flexibility, changing the decisions and plans according to the situation, which can only be done by having a solid bond with the co-teacher.^21^ It places a focus on accountability and responsibility. For teachers to teach simultaneously, collaboration is required, which we explored in this study.

The collaboration starts right from the planning phase; a study described a model used for co-teaching, consisting of three iterative phases: teacher encounter, debriefing, and planning. In our study, there was no such model, but a general planning session would be arranged for teachers to divide the tasks for the session. Planning sessions helped teachers learn much about the topic and gain insight into the innovations and research.^22^ In my study, many participants said planning and conducting the session with senior faculty members was a great learning and professional development chance. Additionally, because co-teaching demands cooperation and compromise, some educators could experience a loss of autonomy. This was discussed by a participant in my study as “the dominating nature of some co-teachers would make you feel uncomfortable”

### Limitations:

Co-teaching, being an innovative tool, is yet not practiced in many regions of the world. Especially in Pakistan, it is only practiced in one course, the ATLS course offered at the Advanced Skill Centre in Karachi. Study’s focus was the participants’ experiences, which were very particular and may not apply to all situations. In the study participants discussed an in-depth exploration of the benefits, but the participants mentioned very few drawbacks

### Recommendations:

- The difficulties of co-teaching and suggesting practical solutions for overcoming them.
- It would also be insightful to look into the long-term effects of co-teaching on teachers’ and students’ professional development.
- Institutions should use co-teaching as a pedagogical method, and instructors should have access to professional development opportunities to improve their co-teaching abilities.
- Institutions should promote collaboration and teamwork among educators to foster an atmosphere supporting co-teaching’s success.

## CONCLUSION

Co-teaching can be used as effective tool for information transfer to students in medical schools. It is so, because in co-teaching two teachers together teach a group of students in a joint space, so if the teacher pair if made of a basic science teacher and clinical teacher or it can be subject specialist and medical educationist. In the integration ladder, the sharing step also supports the concept of co-teaching. Even in the absence of integrated curriculum, co-teaching can do wonders in integrating the basics and clinical sciences. Co-teaching should be implied more in medical sciences for enhanced learning of students. Although it is resourceful in terms of staff required, but it is not impossible. Arranging the timetables in which both teachers are utilized, is one way of overcoming the problems of implementing it.

### Authors’ Contribution:

**AM:** Substantial contributions to study design, acquisition of data, manuscript writing and has given final approval of the version to be published.

**BJ:** Conception of this research and has given final approval of the version to be published.

**MJ:** Substantial contributions to analysis, interpretation of data and has given final approval of the version to be published.

**NA:** Substantial contributions to concept, study design & final write-up, critical review and proof reading.

All authors are agreeing to be accountable for all aspects of the work in ensuring that questions related to the accuracy or integrity of any part of the work are appropriately investigated and resolved.
